# Sodium butyrate activates the K_ATP_ channels to regulate the mechanism of Parkinson's disease microglia model inflammation

**DOI:** 10.1002/iid3.1194

**Published:** 2024-03-19

**Authors:** Ye Xu, Laofu Wen, Yunyi Tang, Zhenqiang Zhao, Miaojing Xu, Tan Wang, Zhibin Chen

**Affiliations:** ^1^ Department of Neurology The First Affiliated Hospital of Hainan Medical University Haikou Hainan China; ^2^ Department of Neurology, Nanfang Hospital Southern Medical University Guangzhou Guangdong China

**Keywords:** ATP‐sensitive potassium channels, microglia, neuroinflammation, Parkinson's disease, sodium butyrate

## Abstract

**Background:**

Parkinson's disease (PD) is a common neurodegenerative disorder. Microglia‐mediated neuroinflammation has emerged as an involving mechanism at the initiation and development of PD. Activation of adenosine triphosphate (ATP)‐sensitive potassium (K_ATP_) channels can protect dopaminergic neurons from damage. Sodium butyrate (NaB) shows anti‐inflammatory and neuroprotective effects in some animal models of brain injury and regulates the K_ATP_ channels in islet β cells. In this study, we aimed to verify the anti‐inflammatory effect of NaB on PD and further explored potential molecular mechanisms.

**Methods:**

We established an in vitro PD model in BV2 cells using 1‐methyl‐4‐phenylpyridinium (MPP^+^). The effects of MPP^+^ and NaB on BV2 cell viability were detected by cell counting kit‐8 assays. The morphology of BV2 cells with or without MPP^+^ treatment was imaged via an optical microscope. The expression of Iba‐1 was examined by the immunofluorescence staining. The intracellular ATP content was estimated through the colorimetric method, and Griess assay was conducted to measure the nitric oxide production. The expression levels of pro‐inflammatory cytokines and K_ATP_ channel subunits were evaluated by reverse transcription–quantitative polymerase chain reaction and western blot analysis.

**Results:**

NaB (5 mM) activated the K_ATP_ channels through elevating Kir6.1 and Kir6.1 expression in MPP^+^‐challenged BV2 cells. Both NaB and pinacidil (a K_ATP_ opener) suppressed the MPP^+^‐induced activation of BV2 cells and reduced the production of nitrite and pro‐inflammatory cytokines in MPP^+^‐challenged BV2 cells.

**Conclusion:**

NaB treatment alleviates the MPP^+^‐induced inflammatory responses in microglia via activation of K_ATP_ channels.

## Graphical abstract

Sodium butyrate (NaB) retrains the activation of the proinflammatory phenotype of microglia by promoting K_ATP_ channel opening through enhancing the expression of Kir6.1 and Kir6.2.

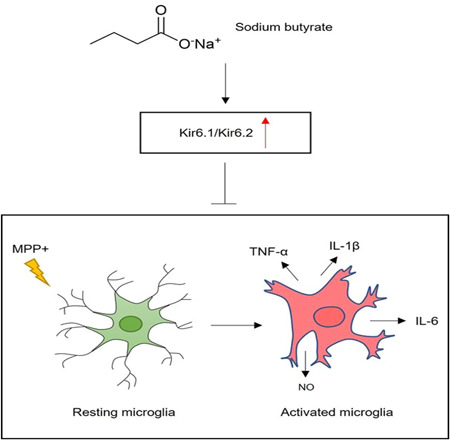

## 1 INTRODUCTION

Parkinson's disease (PD) is the second most common elderly onset neurodegenerative disease manifested by progressive impairment of motor functions such as resting tremors, bradykinesia, and muscle rigidity as well as nonmotor symptoms such as impaired cognition, sleep disorders, depression, constipation, and other autonomic dysfunctions.^1^ The prevalence of PD has doubled in the past 25 years. Global estimates in 2019 showed over 8.5 million individuals living with PD. Disability and death due to PD are also rapidly increasing. In 2019, PD resulted in 5.8 million disability‐adjusted life years, an increase of 81% since 2000, and caused 329,000 deaths, an increase of over 100% since 2000.^2^ Currently, therapies inducing medicines, surgery, and rehabilitation against PD are symptomatic and cannot stop disease progression. Additionally, these therapies have limited effects on severely ill patients, and the long‐term use of clinical drugs has serious adverse effects.^3^, ^4^, ^5^ Therefore, a better understanding of the mechanisms involved in PD and the development of novel effective therapeutic options for PD treatment are urgent.

The pathological studies of PD brains have suggested that the progressive degeneration of dopaminergic (DA) neurons is identified in substantia nigra pars compacta, along with the inclusions of aggregated misfolded α‐synuclein (α‐syn), also known as Lewy bodies.^6^, ^7^ Extracellular α‐syn released by damaged DA neurons leads to abnormal activation of microglia.^8^ Abnormal aggregation of microglia in the substantia nigra of the midbrain has been observed in the patients with PD.^9^ Microglia are the main immune cells in the central nervous system (CNS), and most of them are distributed in the midbrain and maintain the homeostasis of the neuronal microenvironment. In a healthy brain, resting microglia surveil the CNS for potential threats. When the neuronal microenvironment changes, microglia are activated by stimuli and microglia in the reactive state release a wide range of inflammatory factors such as tumor necrosis factor (TNF)‐α, interleukin (IL)−1β, IL‐6, and nitric oxide (NO).^10^, ^11^ The microglia‐mediated neuroinflammation has been implicated in PD pathophysiology.^12^ Inhibition of microglia activation has a profound neuroprotective effect during neuroinflammatory conditions.^13^, ^14^ Therefore, targeting microglia is beneficial by attenuating neuroinflammatory processes in the development of PD.

Adenosine triphosphate (ATP)‐sensitive potassium channels (K_ATP_), a group of vital channels that link the electrical activity of the cell membrane with cell metabolism, are first discovered on the ventricular myocytes of guinea pigs by Noma using the patch‐clamp technique in 1993.^15^ Previous studies have shown that K_ATP_ channels are highly expressed in the substantia nigra, cerebral cortex, hippocampus, and basal ganglia.^16^, ^17^ K_ATP_ channels are hetero‐octamers composed of channel subunits (Kir6.1 or 6.2) and sulfonylurea receptor subunits (SUR1 or SUR2) and are ATP‐binding cassette subfamily members with regulatory activity.^18^, ^19^ They are activated by energy depletion and conduct a weak inwardly rectifying K^+^ current, playing a critical role in the regulation of cellular function by linking cellular metabolism to the electrical activity of cell membranes.^20^, ^21^ The activation of mitochondrial K_ATP_ channels exerts neuroprotective effects on DA neurons by inhibiting glial activation and reducing the products of NO induced by rotenone.^22^ Opening of K_ATP_ channels can inhibit the overproduction of reactive oxygen species and decrease cytochrome c released from mitochondria, thereby preventing MPP^+^‐induced cytotoxicity to primary cultured mesencephalic neurons,^23^ while blockade of K_ATP_ channels with glibenclamide prevents the neuroprotective effects of hydrogen sulfide.^24^ Collectively, activation of K_ATP_ channels can protect DA neurons against damage, and thus K_ATP_ would be a novel target for detection of PD pathogenesis and initiation of effective drugs for PD treatment.

Sodium butyrate (NaB), an inhibitor of histone deacetylase, is shown to reduce the secretion of pro‐inflammatory cytokines by activated microglia,^25^ and alleviate neuroinflammation and neurological damage of PD.^26^ Pretreatment with NaB causes a decrease in inflammatory responses and protects DA neurons from damage in mesencephalic neuron‐glia cultures.^27^ However, the mechanisms by which NaB regulates the microglia‐mediated neuroinflammation in PD remain elusively understood. As reported, NaB inhibits the expression of K_ATP_ channels in islet β cells by downregulating Kcnj11 mRNA expression.^28^ 1‐methyl‐4‐phenylpyridinium ion (MPP^+^), a metabolite of 1‐methyl‐4‐phenyl1‐1, 2,3,6‐tetrahydropyridine can directly activate microglia, and the microglia activation by MPP^+^ can potentiate inflammatory responses and contribute to DA neuronal damage.^29^, ^30^ Therefore, we aimed to investigate the roles and mechanisms of NaB in MPP^+^‐induced BV2 cells. We hypothesized that NaB might inhibit the microglia‐mediated neuroinflammation by regulating the K_ATP_ channels. We believed that NaB could have clinical implications in PD treatment.

## 2 METHODS

### 2.1 Cell culture

The immortalized murine BV2 microglial cell line (Procell) was cultured in Dulbecco's Modified Eagle Medium (Procell) supplemented with 10% fetal bovine serum (Absin) and 1% penicillin/streptomycin (ChemeGen). The incubation was performed in an incubator with 5% CO_2_ at 37°C. Culture medium was replaced once every 24 h and passaged every 2 days.

### 2.2 Cell treatment

The cells at the logarithmic growth stage were seeded in 6‐well plates (1 × 10^6^ cells/well). After reaching 70% confluence, the cells were treated with different concentrations of MPP^+^ (0 μM, 50 μM, 100 μM, 250 μM, 500 μM, 1 mM, and 2 mM) for 24 h. To detect the neuroprotective effects of NaB, the BV2 cells were pretreated with different concentrations of NaB (0, 1.25, 2.5, 5, and 10 mM) for 1 h before MPP^+^ (100 μM) intervention and harvested 24 h after treatment. The morphological changes of BV2 cells were observed using an optical microscope (Olympus). The BV2 cells used as positive control were pretreated with 10 μM pinacidil following treatment with 100 μM MPP^+^.

### 2.3 Cell counting kit‐8 (CCK‐8) assays

The BV2 cells were seeded into 96‐well plates (5 × 10^3^ cells/well) and incubated for 24 h. After the density reaching 50%, the cells were treated with MPP^+^ (0–2 mM) or NaB (0–10 mM) before MPP^+^ (100 μM) intervention for 24 h. Then the CCK‐8 dilution (10 μL/well) was added to each well. After 2 h of incubation, the cell viability was detected with a microplate reader (Molecular Devices) at the wavelength of 450 nm.

### 2.4 Western blot analysis

Total protein was isolated from the BV2 cells using 100 μL precooled RIPA lysis buffer (Sigma‐Aldrich) with 1 μL protease inhibitor (ApexBio Technology) and centrifuged at 14,000 rpm at 4°C for 10 min. A BCA protein assay kit (Yeasen) was prepared for assessment of protein concentration. Protein (20 μg) was boiled in loading buffer, resolved by sodium dodecyl sulfate‐polyacrylamide gel electrophoresis (SDS‐PAGE) gels and transferred onto polyvinylidene difluoride (PVDF) membranes (Millipore). After blocking with 5% nonfat milk, the membranes were incubated overnight with primary antibodies against Kir6.1 (ab241996, 1:1000; Abcam), SUR2A (Bio Excellence International Tech), Kir6.2 (ab79171, 1:1000; Abcam), SUR1 (ab217633, 1:1000; Abcam), and ACTIN (ab8227, 1:2000; Abcam) at 4°C. Then the membranes were incubated with secondary antibodies for 1 h at room temperature. Proteins were visualized by the enhanced chemiluminescence reagent (Yeasen). The immunoblot images were evaluated using ImageJ software.

### 2.5 Detection of ATP

An ATP Bioluminescence Assay Kit (Sigma‐Aldrich) was used to examine the intracellular ATP levels according to the manufacturer's instructions. Briefly, the cells were lysed with the cell lysis reagent containing 50 μL of the luciferase reagent. The chemiluminescence of the samples was detected using a microplate reader. The ATP concentrations of the samples were calculated using ATP standard and normalized to the protein concentrations of the samples that were determined by BCA assays.

### 2.6 Immunofluorescence

The BV2 cells were fixed with 4% paraformaldehyde (ChemeGen) and washed by phosphate buffer saline (PBS; 5 min/time, three times). Subsequently, the cells on glass slides were permeabilized using 0.1% Triton X‐100 (Beyotime) for 20 min, and then blocked with 5% bovine serum albumin (Beyotime) for 1 h at room temperature. The cells were incubated with the primary antibody against Iba‐1 at 4°C overnight. Thereafter, the cells were washed using PBS three times and each time lasted 5 min, and then incubated with corresponding secondary antibody for 1 h at room temperature. Nuclei were visualized by DAPI (Solarbio). The fluorescence signals were imaged with a fluorescence microscope (Olympus). The images were analyzed via ImageJ software and the number of Iba‐1‐positive cells were quantified. Data were an average of 10 randomly selected regions.

### 2.7 Reverse transcription–quantitative polymerase chain reaction (RT‐qPCR)

Total RNA was separated from BV2 cells using TRIzol reagent (Absin), and total RNA (1 μg) was reverse‐transcribed in a reaction mixture containing 1 U RNase inhibitor, 500 ng random primer, 3 mM MgCl_2_, 0.5 mM dNTP, 1X RT buffer, and 10 U reverse transcriptase (Promega). The cDNA served as a template for the PCR reaction, which was performed using Go Taq polymerase (Promega) and primers. The RT‐qPCR was carried out on the thermal cycler (Bio‐Rad) and on the ABI PRISM 7000 Sequence Detection System (Applied Biosystems) using Sensi FAST SYBR Hi‐ROX Mix (Bioline). GAPDH was used as an endogenous reference for mRNA detection. Data were calculated using the 2^−△△CT^ method.^31^ The experimental operating conditions were 95°C for 10 min, 95°C for 5 s, and 60°C for 60 s for 40 cycles. The primer sequences used in this study are listed in Table 1.

**Table 1:** Sequences of primers used for reverse transcription–quantitative PCR.

Gene	Sequence (5′ → 3′)
IL‐6 forward	CTGCAAGAGACTTCCATCCAG
IL‐6 reverse	AGTGGTATAGACAGGTCTGTTGG
TNF‐α forward	CAGGCGGTGCCTATGTCTC
TNF‐α reverse	CGATCACCCCGAAGTTCAGTAG
IL‐1β forward	GAAATGCCACCTTTTGACAGTG
IL‐1β reverse	TGGATGCTCTCATCAGGACAG
GAPDH forward	AATGGATTTGGACGCATTGGT
GAPDH reverse	TTTGCACTGGTACGTGTTGAT

Abbreviations: IL, interleukin; PCR, polymerase chain reaction; TNF‐α, tumor necrosis factor‐α.

### 2.8 Determination of NO production

NO production was determined by measuring nitrite production as previously described.^32^, ^33^ Briefly, Griess reagent (Beyotime) was freshly prepared by mixing equal volumes of 0.1% napthylethylenediamine dihydrochlroride and 1% sulfanilamide in 5% phosphoric acid and then added to spent culture media for 5 min. These mixtures were then evaluated for absorbance at 540 nm using a spectrophotometer (Molecular Devices), and NO was quantified using a sodium nitrite (1–80 μM) standard curve.^34^

### 2.9 Statistics analysis

All data were averaged from three independent experiments. Two observers who were blinded to the experimental design participated in statistics analysis. All results were analyzed by GraphPad Prism 6.0 (GraphPad Inc.) and reported as the mean ± standard deviation (SD). One way analysis of variance followed by Tukey's post hoc analysis was used for statistical analysis. *p* < .05 was considered as statistically significant.

## 3 RESULTS

### 3.1 The cytotoxicity of MPP^+^ to BV2 cells

**Figure 1:**
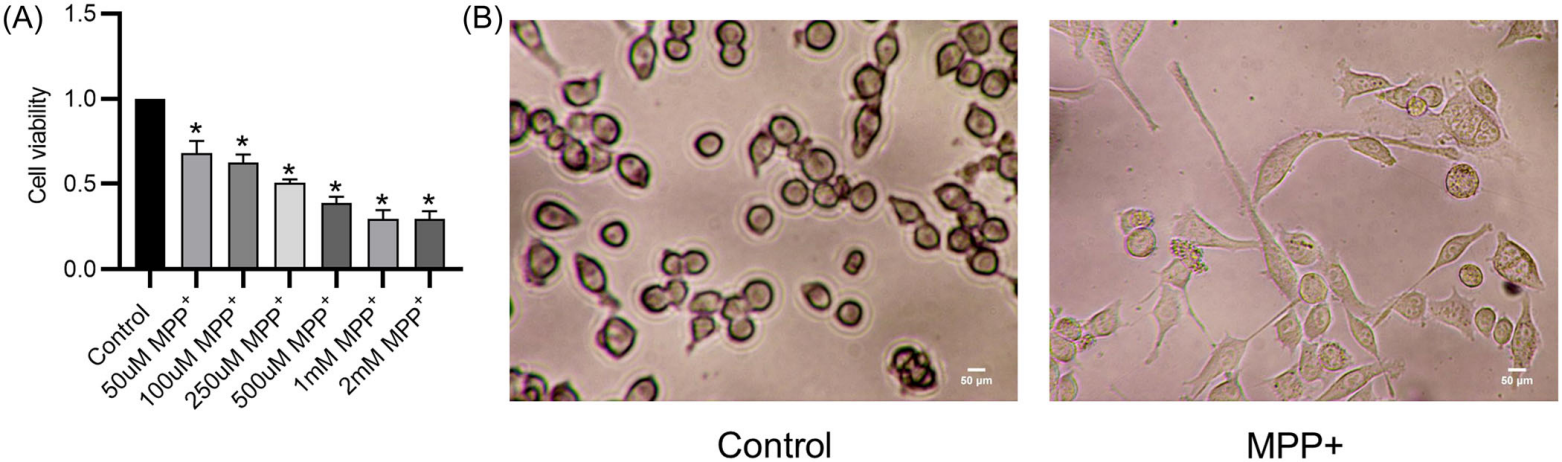
The cytotoxicity of MPP^+^ to BV2 cells. (A) The effects of MPP^+^ (0–2 mM) on cell viability were detected by CCK‐8 methods. (B) The morphological changes of BV2 cells with or without MPP^+^ treatment were observed via an optical microscope. *p < .05.

Cell viability was detected via the CCK‐8 method, and the results showed that the BV2 cells treated with different concentrations (50 μM–2 mM) of MPP^+^ exhibited decreased cell viability (Figure 1A). According to the influence of MPP^+^ on cell viability and the drug concentration used in previous studies,^35^, ^36^ 100 μM was chosen as the effective treatment concentration used for subsequent experiments. The cell morphologies of untreated or MPP^+^‐treated BV2 cells were observed via an optical microscope. The untreated BV2 cells were in a resting state and presented small round or spindle cell bodies with a few synapses. After treatment with MPP^+^, the BV2 cells were activated characterized by thick and short cell bodies with branching or amebic synapses (Figure 1B). These results suggest that MPP^+^ causes cytotoxicity to BV2 cells and leads to activation of BV2 cells.

### 3.2 The protective effects of NaB against MPP^+^‐induced damage to BV2 cells

**Figure 2:**
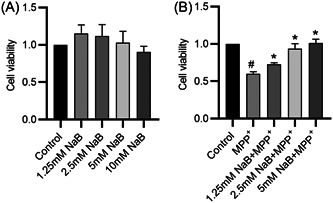
The protective effects of sodium butyrate (NaB) against MPP^+^‐induced damage to BV2 cells. (A) The effects of NaB (0–10 mM) on cell viability. (B) The viability of BV2 cells in the control, the MPP^+^, the 1.25 mM NaB + MPP^+^, the 2.5 mM NaB + MPP^+^, and 5 mM NaB + MPP^+^ groups. ^#^ *p* < .05 compared to control group; *p < .05 compared to MPP^+^ group.

There was no significant difference in cell viability between the groups of BV2 cells treated with different concentrations of NaB (0–10 mM), suggesting that NaB had no cytotoxicity to BV2 cells at the concentrations of 0 mM to 10 mM (Figure 2A). The CCK‐8 results also revealed that the viability of BV2 cells treated with 100 μM MPP^+^ was significantly decreased, whereas pretreatment with 1.25, 2.5, and 5 mM NaB rescued the decreased cell viability, and the promoting effect of NaB on cell viability was optimal at the concentrations of 5 mM (Figure 2B). Therefore, the cells pretreated with 5 mM NaB before 100 μM MPP^+^ were used for subsequent studies.

### 3.3 The effects of NaB on the expression level of K_ATP_ channel subunits at different time points

**Figure 3:**
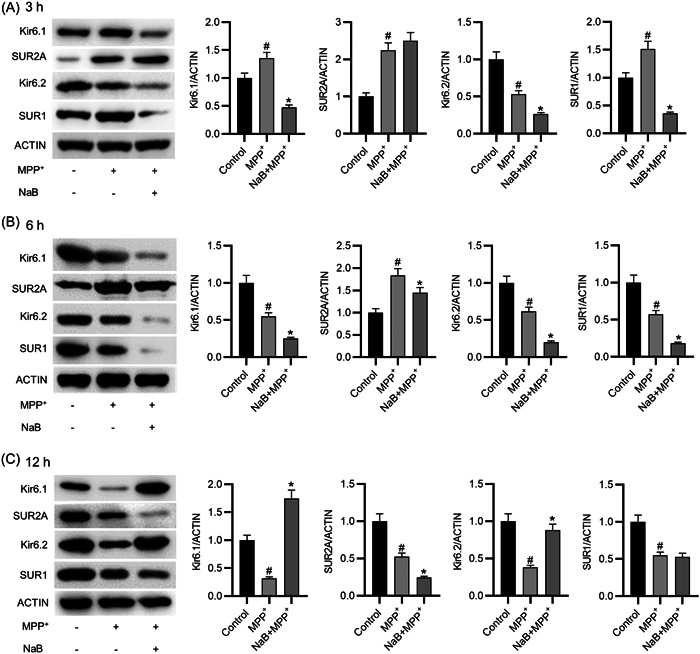
The effects of sodium butyrate (NaB) on the expression level of K_ATP_ channel subunits at different time. (A) The protein levels of Kir6.1, SUR2A, Kir6.2, and SUR1 after 3‐h treatment of NaB. (B) The protein levels of Kir6.1, SUR2A, Kir6.2, and SUR1 after 6‐h treatment of NaB. (C) The protein levels of Kir6.1, SUR2A, Kir6.2, and SUR1 after 12‐h of treatment. ^#^ *p* < .05 compared to control group; *p < .05 compared to MPP^+^ group.

Total protein isolated from the BV2 cells pretreated with NaB for 3, 6, or 12 h was collected for western blot analysis. The western blot analysis results demonstrated that compared to those in the corresponding MPP^+^ groups, the protein levels of Kir6.1, Kir6.2, and SUR1 in the NaB (3 h) + MPP^+^ and NaB (6 h) + MPP^+^ groups were significantly decreased, while the MPP^+^ (12 h) group exhibited significantly elevated protein levels of Kir6.1 and Kir6.2 and unaltered protein level of SUR1, suggesting that the expression levels of Kir6.1 and Kir6.2 were increasing with time. In parallel, the protein level of SUR2A in both NaB (3 h) + MPP^+^ and NaB (12 h) + MPP^+^ groups were not significantly different from that in the corresponding MPP^+^ groups, while its protein level was significantly decreased in the NaB (6 h) + MPP^+^ group than that in the corresponding MPP^+^ group (Figure 3). Collectively, these results demonstrate that NaB activates the K_ATP_ channels through upregulation of Kir6.1 and Kir6.1 expression.

### 3.4 The effects of NaB on K_ATP_ channels and microglia activation

**Figure 4:**
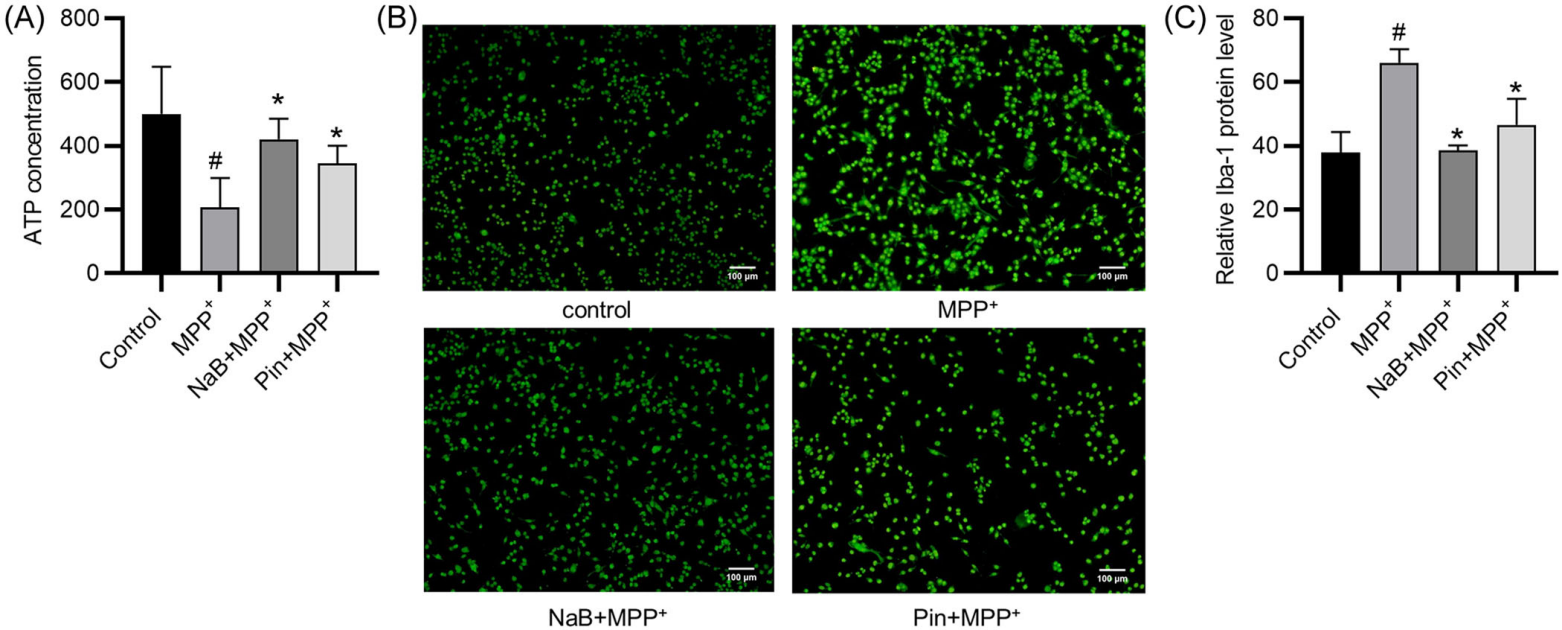
Sodium butyrate (NaB) facilitates the activation of K_ATP_ on microglia and inhibits the activation of microglia. (A) The adenosine triphosphate (ATP) content in the control, the MPP^+^, the NaB + MPP^+^, and the Pin + MPP^+^ groups. (B and C) Immunofluorescence staining of Iba‐1 expression after indicated treatment. ^#^ *p* < .05 compared to control group; *p < .05 compared to MPP^+^ group.

As shown in Figure 4A, the MPP^+^‐treated BV2 cells exhibited significantly lower ATP content than the negative control, while NaB pretreatment prevented the MPP^+^‐induced reduction in ATP content, and the effect of NaB was similar to that of pinacidil (Figure 4A). Low level of intracellular ATP content is an important modular of K_ATP_ channel opening.^37^ Therefore, the results showed that NaB reversed the decreased ATP content in BV2 cells via activation of K_ATP_ channels. Moreover, the results of immunofluorescence staining on Iba‐1 demonstrated that the number of Iba‐1 positive cells were increased in the MPP^+^ group compared with the control group, whereas NaB or pinacidil treatment attenuated the MPP^+^‐induced increase (Figure 4B). These results suggest that both NaB and pinacidil (a K_ATP_ opener) can prevent the MPP^+^‐induced microglia activation.

### 3.5 NaB prevents the production of NO and pro‐inflammatory cytokines in MPP^+^‐treated BV2 cells

**Figure 5:**
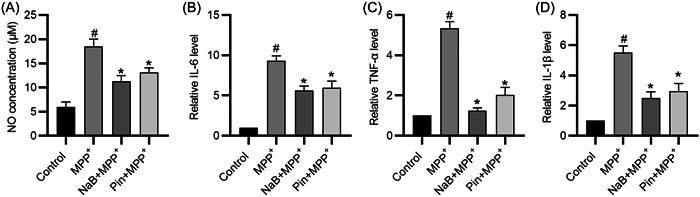
Sodium butyrate (NaB) prevents the production of nitric oxide (NO) and pro‐inflammatory cytokines by MPP^+^‐treated BV2 cells. (A) NO concentrations in the control, the MPP^+^, the NaB + MPP^+^, and the Pin + MPP^+^ groups. (B–D) Reverse transcription–quantitative polymerase chain reaction of interleukin (IL)‐6, tumor necrosis factor (TNF)‐α, and IL‐1β expression levels. ^#^ *p* < .05 compared to control group; *p < .05 compared to MPP^+^ group.

The MPP^+^‐treated BY2 cells produced more NO than the negative control, whereas NaB pretreatment reversed the MPP^+^‐induced promotion, and the effect of NaB was similar to that of the pinacidil (Figure 5A). The results of RT‐qPCR showed that NaB or pinacidil pretreatment inhibited the MPP^+^‐induced promotion in the mRNA levels of IL‐6, TNF‐α, and IL‐1β in BV2 cells (Figure 5B–D). In conclusion, both NaB and pinacidil pretreatments attenuate the MPP^+^‐induced inflammation in BV2 cells.

## 4 DISCUSSION

In the early 1980s, activated microglial infiltrations were observed by MvGeer in the substantia nigra of the postmortem PD brain.^38^ Henceforth, there are growing evidence that microglia‐mediated neuroinflammation is a critical player in the pathogenesis and progression of PD.^39^, ^40^, ^41^ Activated microglia is suggested to release pro‐inflammatory cytokines and neurotoxic factors that exacerbate DA neuronal degeneration in PD.^42^ MPP^+^ can cause microglia activation and stimulate the release of pro‐inflammatory cytokines.^43^ Therefore, in the current study, we treated BV2 cells with MPP^+^ to induce PD‐like conditions in vitro. NaB has exhibited antiapoptotic properties in neurodegenerative disorders and can cause α‐syn degradation, inhibit overactivation of glial cells and inflammatory responses and ameliorate motor deficits in PD models.^44^, ^45^, ^46^, ^47^ In this study, we have explored the underlying mechanisms by which NaB exerts protective effects against PD‐like conditions. NaB treatment inhibits the microglia‐mediated neuroinflammation by activating the K_ATP_ channels as indicated by following findings: (a) NaB treatment restrained the MPP^+^‐induced impairment in the viability of BV2 cells; (b) NaB treatment activated the K_ATP_ channels by upregulating Kir6.1 and Kir6.1 expression; (c) NaB and K_ATP_ opener prevented the MPP^+^‐induced microglia activation; (d) NaB and K_ATP_ opener inhibited inflammatory responses in MPP^+^‐treated BV2 cells.

After treatment with 100 μM MPP^+^, the morphology of BV2 wells changed from resting state to activated state. Iba‐1 is recognized as a sensitive marker to evaluate the activation of microglia.^48^ Iba‐1 is expressed on both resting and activated microglia, but the activated microglia exhibit more Iba‐1.^49^ In the current study, we found significantly increased number of Iba‐1 positive cells after MPP^+^ treatment, suggesting that MPP^+^ significantly activated BV2 cells, and PD‐like conditions were successfully induced in MPP^+^‐treated BV2 cells. The suppressive functions of NaB in microglia activation have been well documented.^50^, ^51^, ^52^ In the current study, we found that pretreatment with NaB significantly attenuated the immunoreactivity of Iba‐1, indicating that NaB prevented the activation of microglia.

Microglia‐mediated neuroinflammation is suggested to be a common feature of neurodegenerative diseases, including PD.^53^ Activation of microglia induces the release of large numbers of pro‐inflammatory cytokines such as TNF‐α, IL‐6, IL‐1β, leading to progressive neuron loss.^54^ NO released from microglia is an inflammatory mediator, and its overproduction induces neurotoxicity.^55^ As reported, NaB plays an anti‐inflammatory role in the overnutrition‐induced activated microglia.^50^ Additionally, NaB treatment results in neurogenesis in the damaged ipsilateral side and reduces the neuronal apoptosis via inhibition of inflammation.^56^ The current study demonstrated that NaB treatment limited the promotion in the production of NO, TNF‐α, IL‐6, and IL‐1β, suggesting that NaB might inhibit the MPP^+^‐induced microglia activation to exert anti‐inflammatory effects.

The functional K_ATP_ channels are formed by Kir6x (Kir6.1 and Kir6.2) and SUR subunits (SUR1, SUR2A, and SUR2B) which are ATP‐binding cassette subfamily members with regulatory activity.^19^, ^57^ The K_ATP_ opening is dependent on ATP content that when ATP content decreases, the K_ATP_ channels allow the passage of K^+^, thereby hyperpolarizing the membranes of microglia in the CNS, decreasing cell excitability and attenuating ATP assumption.^58^ Opening of the K_ATP_ channels can inhibit rotenone‐induced neuroinflammation and glia activation.^59^ In the current study, we found that BV2 cells expressed SUR1, SUR2A, Kir6.1, and Kir6.2, indicating the presence of K_ATP_ channels in BV2 cells. NaB is reported to reduce the expression of Kcnj11 (encoding K_ATP_ channels) in islet β cells.^28^ The current study revealed that NaB significantly reversed the MPP^+^‐induced reduction in ATP content in BV2 cells and upregulated the expression levels of Kir6.1 and Kir6.2 levels and had no significant influence on the expression levels of SUR1 and SUR2A, suggesting that NaB regulated the K_ATP_ channels through upregulation of Kir6.1 and Kir6.2. Additionally, opening of K_ATP_ channels by pinacidil also inhibited microglia activation and inflammatory responses caused by MPP^+^.

In conclusion, this study demonstrated that NaB treatment protected against MPP^+^‐induced neuroinflammation by inhibiting microglia activation. Its underlying mechanisms may be involved in the activation of K_ATP_ channels by upregulating Kir6.1 and Kir6.2 expression. This study might confer novel therapeutic thoughts in pathological reaction of PD.

To be honest, there are limitations to this study. First, Du et al.^60^ reported that Kir6.1 deficiency exacerbates DA neuron degeneration by promoting excessive microglia activation in mice with PD, and Kir6.2 knockout reduces astrocytic activation and restores DA neuron death.^61^ Further detection of Kir6.1 and Kir6.2 effects on microglia is required. Second, in vivo experiments are lacked in this study.

## AUTHOR CONTRIBUTIONS

Ye Xu and Laofu Wen conceived and designed the experiments. Miaojing Xu, Laofu Wen, Yunyi Tang, Zhenqiang Zhao, Ye Xu, Tan Wang, and Zhibin Chen carried out the experiments. Ye Xu, Miaojing Xu, Laofu Wen, Tan Wang, and Zhibin Chen analyzed the data. Ye Xu, Miaojing Xu, Laofu Wen, Tan Wang, and Zhibin Chen drafted the manuscript. All authors agreed to be accountable for all aspects of the work. All authors have read and approved the final manuscript.

## CONFLICT OF INTEREST STATEMENT

The authors declare no conflict of interest.

## Data Availability

The datasets used or analyzed during the current study are available from the corresponding author on reasonable request.
